# The Ophthalmic Year Book

**Published:** 1905-05

**Authors:** 


The Ophthalmic Year Book. A Digest of the Literature of Ophthalmology for the year 1903. By Edward Jackson, A. M., M. D., Emeritus Professor of Diseases of the Eye in the Philadelphia Polyclinic. Octavo, pp. 260. With 45 illustrations. Denver: The Herrick Book and Stationery Company. 1904.

The author of this digest is an ophthalmologist of experience and well knows how to winnow the wheat from the chaff of ophthalmological literature. In this year book for 1903, the first volume of a proposed series, he has made abstracts and digests from the ophthalmological literature of the world, and has systematically arranged them in such a manner as to present to the reader, at a glance, the essential progress that has been made during the previous year in this department of practice. His aim has been, as he says in the preface, "First, a critical digest of the most important literature of the past year, judging it from the standpoint of the ophthalmic surgeon,—not extracts or an outline of that literature; but the important things, given sufficiently in detail to make them applicable in practice. Second, a list of the more important original communications appearing during the year. The design is to help the practitioner in this branch to keep up with the progress of his specialty; and with this to supply a series of volumes which will bring him in touch with all the more important recent communications upon any subject."

To the ophthalmologist who desires to have in hand a good digest of the ophthalmological literature of 1903, there is nothing in the English language that will better serve the purpose.

